# Rapid, actionable diagnosis of urban epidemic leptospirosis using a pathogenic *Leptospira lipL32*-based real-time PCR assay

**DOI:** 10.1371/journal.pntd.0005940

**Published:** 2017-09-15

**Authors:** Irina N. Riediger, Robyn A. Stoddard, Guilherme S. Ribeiro, Sueli M. Nakatani, Suzana D. R. Moreira, Irene Skraba, Alexander W. Biondo, Mitermayer G. Reis, Alex R. Hoffmaster, Joseph M. Vinetz, Albert I. Ko, Elsio A. Wunder

**Affiliations:** 1 Molecular Biology Section, Central Laboratory of the State of Paraná, Curitiba, Paraná Brazil; 2 National Center for Emerging and Zoonotic Infectious Diseases, Centers for Disease Control and Prevention, Atlanta, Georgia, United States of America; 3 Gonçalo Moniz Institute, Oswaldo Cruz Foundation, Brazilian Ministry of Health, Salvador, Bahia, Brazil; 4 Institute of Collective Health, Federal University of the State of Bahia, Salvador, Bahia, Brazil; 5 Hospital das Clínicas, Federal University of the State of Paraná, Curitiba, Paraná, Brazil; 6 Department of Veterinary Medicine, Federal University of the State of Paraná, Curitiba, Paraná, Brazil; 7 Division of Infectious Diseases, Department of Medicine, University of California San Diego School of Medicine, La Jolla, California, United States of America; 8 Department of Epidemiology of Microbial Diseases, Yale School of Public Health, New Haven, Connecticut, United States of America; University of Texas Health Science Center at Houston McGovern Medical School, UNITED STATES

## Abstract

**Background:**

With a conservatively estimated 1 million cases of leptospirosis worldwide and a 5–10% fatality rate, the rapid diagnosis of leptospirosis leading to effective clinical and public health decision making is of high importance, and yet remains a challenge.

**Methodology:**

Based on parallel, population-based studies in two leptospirosis-endemic regions in Brazil, a real-time PCR assay which detects *lipL32*, a gene specifically present in pathogenic *Leptospira*, was assessed for the diagnostic effectiveness and accuracy. Patients identified by active hospital-based surveillance in Salvador and Curitiba during large urban leptospirosis epidemics were tested. Real-time PCR reactions were performed with DNA-extracted samples obtained from 127 confirmed and 23 unconfirmed cases suspected of leptospirosis, 122 patients with an acute febrile illness other than leptospirosis, and 60 healthy blood donors.

**Principal findings:**

The PCR assay had a limit of detection of 280 *Leptospira* genomic equivalents/mL. Sensitivity for confirmed cases was 61% for whole blood and 29% for serum samples. Sensitivity was higher (86%) for samples collected within the first 6 days after onset of illness compared to those collected after 7 days (34%). The real-time PCR assay was able to detect leptospiral DNA in blood from 56% of serological non-confirmed cases. The overall specificity of the assay was 99%.

**Conclusions:**

These findings indicate that real-time PCR may be a reliable tool for early diagnosis of leptospirosis, which is decisive for clinical management of severe and life-threatening cases and for public health decision making.

## Author summary

In this study, we describe the performance testing and diagnostic accuracy assessment of a previously described real-time PCR assay aiming at the validation for early diagnosis of human leptospirosis. We found that diagnostic sensitivity was higher for EDTA whole blood samples (61%) than for serum samples (29%). We demonstrated that the molecular diagnosis was optimal when performed with EDTA whole samples collected within the first 6 days with symptoms, condition in which the sensitivity was 86%. The real-time PCR assay was able to detect leptospiral DNA in EDTA whole blood from 56% of serological non-confirmed cases for whom a convalescent sample was not available. The test overall specificity was 99%. Our findings demonstrate that real-time PCR is a reliable diagnostic tool suited for early diagnosis, and its results could support early medical interventions that could have a decisive impact on clinical outcome and thus decrease both the severity and fatality of cases.

## Introduction

Leptospirosis is the most widespread zoonosis worldwide, particularly during the rainy season in tropical developing countries [1], where uncontrolled growth of slum communities predispose humans to rodent-borne transmission [2, 3]. The global impact of leptospirosis is substantial, affecting a conservatively estimated 1 million individuals per year with case fatality rates of 5–10% [4, 5].

The infection, usually acquired by indirect contact with urine from animal reservoirs, leads to clinical manifestations which range from subclinical infections to life-threatening syndromes [6]. Most leptospirosis symptoms are non-specific and may be shared with other tropical infectious diseases, posing a challenge to clinical differential diagnosis [1].

Laboratory testing is typically based on well-established serological and microbiological techniques [1, 6]. Serological methods such as the microscopic agglutination test (MAT), which is the serological gold standard method [7], ELISA, and dipstick-type rapid diagnostic tests based on *L*. *biflexa* antigen have low sensitivity during the acute phase of illness [8]. In endemic regions, those serological methods may not distinguish current from previous infection. Culture does not contribute to acute diagnosis because *Leptospira* grow slowly over weeks [9].

The demand for early diagnosis has driven the development of several PCR-based assays since direct detection of *Leptospira* nucleic acids may precede antibody detection [6]. A number of real-time PCR-based methods have been described for leptospirosis diagnosis [10–21], some of which have been evaluated in clinical settings [10, 12, 15, 17, 19–21]. However, diagnostic studies on large populations using real-time PCR methods [21, 22] have not compared the molecular diagnostic performance of EDTA whole blood (EWB) and serum [23]. Additionally, most subjects were selected from low incidence areas [24], leaving real-time PCR diagnostic applicability uncertain in high-risk regions.

This study aimed at clinically validating a previously described *lipL32-*based real-time PCR assay [16] in two geographically distinct regions in Brazil where epidemic leptospirosis is common, and determining whether this test provided actionable results for clinical care and public health control measures.

## Methods

### Ethics statement

The study protocol was approved by the Institutional Review Board (IRB) committees of Oswaldo Cruz Foundation, Brazilian Ministry of Health (CONEP 36245314.9.0000.0040); and of Hospital das Clínicas of the Federal University of the State of Paraná (CONEP 0046.1.208.208–09). Written informed consent, approved by the IRB committees, was obtained from all patients, controls, healthy individuals who donated blood to Hospital das Clínicas Blood Bank and a leptospirosis naïve donor. Adult subjects provided informed consent, and a parent or guardian of child participants provided informed consent on their behalf. All the samples were anonymized for use in this study.

### Human subject enrollment

Candidates for enrollment in this study were prospectively and consecutively identified from October 2007 to July 2009, in two contexts and sites: (i) during active hospital-based surveillance in the city of Salvador, northeastern Brazil and (ii) from those who sought medical treatment at Hospital de Clínicas of the Federal University of the State of Paraná or at one of the 132 Municipal Health Units in the city of Curitiba, southern Brazil.

**Fig 1:**
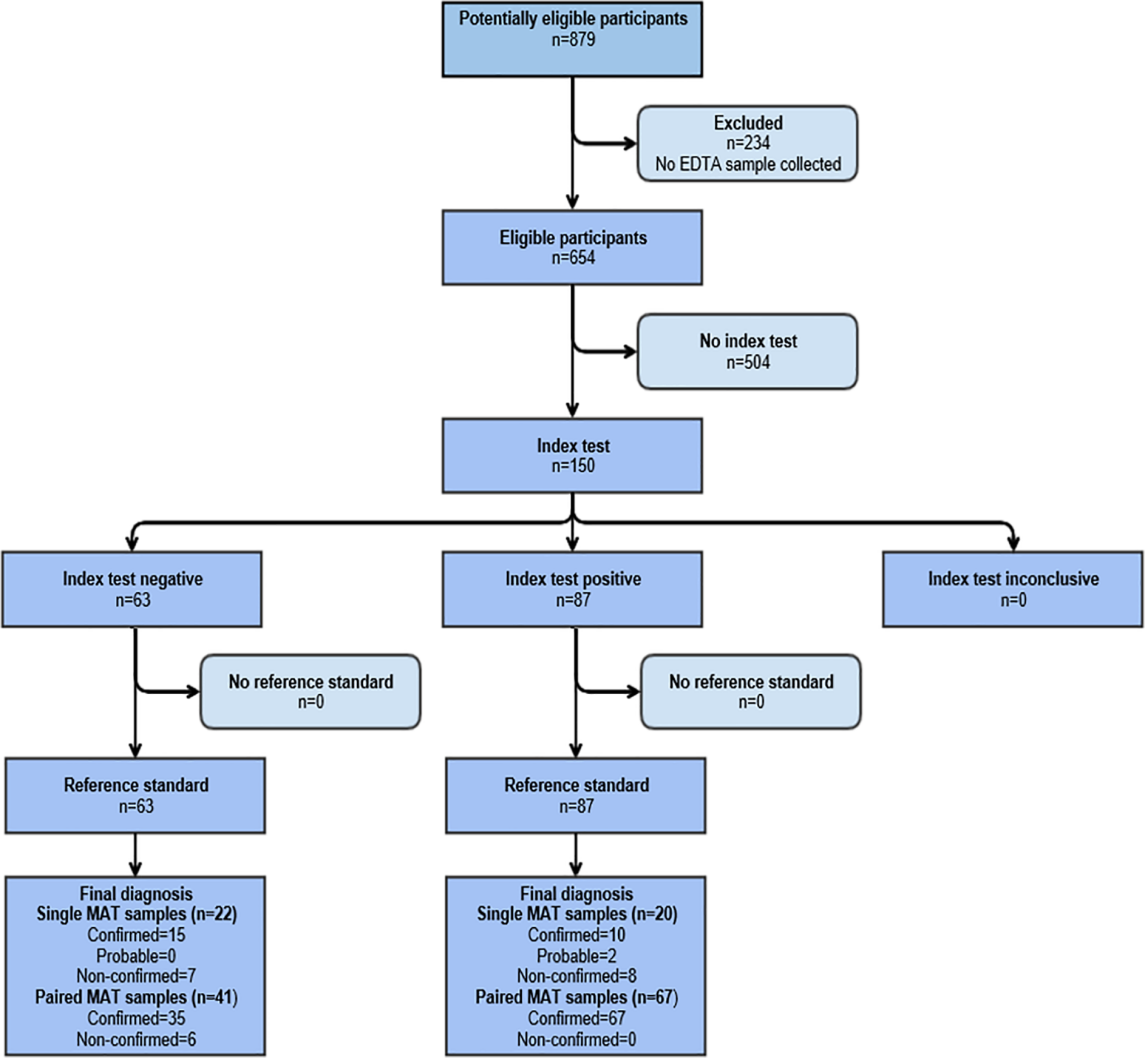
Standards for Reporting of Diagnostic Accuracy (STARD) flow chart of the 150 suspected leptospirosis cases enrolled in the study. Patients were selected in two Brazilian cities: Curitiba (A) and Salvador (B).

Inclusion criteria were based on clinical suspicion of leptospirosis, i.e. specific findings at physical examination or characteristic manifestations of leptospirosis [2]. Blood samples were drawn at admission for *Leptospira* culture, serological testing and molecular diagnosis. For most patients, a convalescent serum sample was obtained approximately two weeks later. Serum and EDTA whole blood (EWB) samples were stored at -20°C and -80°C, respectively, until being processed. Reference tests were executed by trained personnel that did not have access to patients’ clinical information and that were blinded to the outcome of the qPCR tests. The maximum time interval between the performance of the reference and index tests was three months. For culture, 100 μL of fresh blood were inoculated into tubes containing liquid and semisolid Ellinghausen, McCullough, Johnson and Harris (EMJH) media. Cultures were incubated at 30°C and inspected weekly by dark-field microscopy for up to 3 months. At Curitiba, MAT was performed according to standard criteria [25] using a 22 reference strain panel. At Salvador, we used a reduced panel of seven reference strains for MAT since previous studies have shown that on this site, 99% of confirmed cases are due to serovar Copenhageni [2]. Additional information on the strains used for MAT are provided in the S1 Table (S1 Table). Laboratory confirmation was based on isolation of *Leptospira* from a blood sample and/or a positive MAT result. The criteria for the positive MAT was seroconversion or fourfold rise in MAT titers between paired serum samples, or MAT titer ≥ 1:800 in a single sample. Probable cases were defined as all individuals not included as confirmed which had a MAT titer ≥ 1:100 and <1:800 in one or more serum samples. A total of 150 clinically suspected leptospirosis cases were enrolled in the study. In Salvador, samples were collected from 85 laboratory-confirmed, 1 probable and 13 non-confirmed cases (n = 99). In Curitiba, samples were obtained from 42 laboratory-confirmed patients, 1 probable case and 8 non-confirmed patients (n = 51). Standards for Reporting of Diagnostic Accuracy (STARD) flow chart and checklist are provided as Fig 1 and S2 Table, respectively. Negative controls consisted of serum and EWB samples obtained from 122 hospitalized patients diagnosed with acute febrile illnesses (AFI) other than leptospirosis (65 cases from Salvador and 57 from Curitiba), and from 60 healthy controls (blood bank donors).

### Assay development

Four independent spiking experiments were performed using *L*. *interrogans* serovar Copenhageni strain Fiocruz L1-130. Bacterial concentration was determined using a Petroff-Hausser counting chamber (Hausser Scientific, Horsham, PA, USA). Bacteria were counted three times and the mean count was used in subsequent experiments.

Blood from a leptospirosis naive donor (serologically and PCR negative) was collected in EDTA and SST vacuum tubes (Vacutainer K_2_ EDTA and Vacutainer SST tubes, BD Diagnostics, Sparks, MD, USA). Leptospires were spiked into EWB, uncoagulated blood in SST tubes and ultrapure water to obtain a final concentration of 1 × 10^6^ leptospires/mL. After clotting, the spiked SST tube was centrifuged at 1,000 × *g* for 15 min to obtain the corresponding serum sample. Spiked specimens were serially 10-fold diluted down to 1 × 10^0^ leptospires/mL. Simultaneously processed DNA extracts of unspiked aliquots of ultrapure water, EWB and serum were used as negative controls.

### DNA extraction

The DNeasy Blood & Tissue kit (QIAGEN, Valencia, CA, USA) was used for DNA extraction. DNA was extracted from 200 μL of each specimen following manufacturer’s instruction, except for using 100 μL of AE buffer for DNA elution, performed after an incubation of 10 minutes at room temperature prior to the last centrifugation.

### Calibration curve

Genomic DNA (gDNA) obtained from strain L1-130 was quantified using a spectrophotometer (ND-1000 Nanodrop, Nanodrop Technologies, Wilmington, DE, USA). Genomic equivalents (GEq) were calculated based on a genome size of 4,627 Mb of *L*. *interrogans* serovar Copenhageni strain Fiocruz L1-130 [26]. Calibrators were prepared upon adjustment of DNA concentration to 1 × 10^7^ GEq/5 μL followed by 10-fold serial dilution down to 10^0^ GEq/5 μL.

### Real-time PCR assay

Real-time PCR tests were executed by trained personnel that were blinded to the outcome of the reference tests. TaqMan real-time PCR assays were carried out using the ABI 7500 Real-Time PCR System (Applied Biosystems, Foster, CA, USA) (16). The reaction mix consisted of 12.5 μL of Platinum Quantitative PCR SuperMix-UDG (Invitrogen, Carlsbad, CA, USA), 500 nM of forward and reverse primers, 100 nM of probe, 5 μL of DNA extract and ultrapure water to a final volume of 25 μL. The human *RPP30* gene was used as an endogenous internal control, amplified using the same reaction mix described above for the *lipL32* gene except for the use of 200 nM of primers [16]. For both targets, the amplification protocol was 2 min at 50°C and 10 min at 95°C, then 45 cycles of 95°C for 15 s and annealing/extension at 60°C for 1 min.

No-template controls (5 μL of ultrapure water added instead of DNA) and a positive control were included in each run. Samples in which *lipL32* and *RPP30* were detected within 40 and 32 PCR cycles, respectively, were considered positives.

### Real-time PCR performance test

Consecutive calibration curves were used to assess the assay’s efficiency, linear dynamic range and precision (repeatability and reproducibility). Spiked and serially diluted biological matrices were used to evaluate trueness (accuracy). The lower limit of detection (LLOD) was determined by testing 24 replicates of serial gDNA dilutions with concentrations ranging from 20 GEq/5 μL to 1 GEq/5 μL.

### Statistical analysis

Linear regression of log-transformed data was used to calculate the slope and the regression coefficient (R^2^) used in the efficiency, linear dynamic range and trueness analysis. Analytical sensitivity (LLOD targeting a 95% hit-rate) was estimated by probit regression analysis (PASW Statistics for Windows v. 18, SPSS Inc., Chicago, IL, USA). Statistical significance of mean differences of demographic and clinical characteristics of patients was assessed by either Student’s unpaired *t* test (quantitative variables) or chi-square test (categorical variables). Univariate analysis of variance was used to compare the means of log-converted GEq amounts of *Leptospira* in spiked water, EWB and serum samples, followed by Tukey-Kramer multiple comparison test. Statistical significance was assumed for *p-*values <0.05. Clinical sensitivity and 95% confidence intervals (CI) were inferred whenever appropriate and samples obtained from negative controls were used to determine clinical specificity.

The present analysis adhered to STARD [27, 28] and Minimal Information for Publication of Quantitative real-time PCR Experiments (MIQE) [29].

## Results

### Analytical performance tests

Amplification of the calibrators was linear through eight orders of magnitude, with a regression coefficient (R^2^) of 0.9944 (S1 Fig) and an overall amplification efficiency calculated to be 96.0% [30].

Data from successive amplifications of the calibration curve used for the precision analysis is shown in Table 1. The overall precision showed a cycle threshold (Ct) coefficient of variation (CV) range from 0.9 to 12.5%, with higher CVs at 1 GEq/reaction (Table 1). According to the probit regression analysis, a 95% hit-rate LLOD would be achieved at 2.8 GEq/5 μL.

**Table 1:** Precision analysis of the *lipL32* real-time PCR assay.

Nominal Conc (GEq/rxn)	Repeatability^a^				Reproducibility^b^			
	Mean Conc. (GEq/rxn)	Mean Ct^c^	SD (Log_10_)	CV (%)	Mean Conc. (GEq/rxn)	Mean Ct	SD (Log_10_)	CV (%)
1	2	38.44	0.17	68.44	2	38.64	0.16	83.80
10	12	36.62	0.11	10.61	12	36.61	0.13	12.51
100	98	33.06	0.07	3.51	111	32.86	0.09	4.28
1,000	935	29.48	0.07	2.20	1,014	29.38	0.09	3.12
10,000	10,191	25.91	0.06	1.53	10,261	25.89	0.05	1.28
100,000	103,669	22.50	0.07	1.34	96,961	22.57	0.08	1.53
1,000,000	1,134,389	19.00	0.07	1.10	1,095,684	19.03	0.05	0.90
10,000,000	11,187,790	15.65	0.06	0.86	10,497,559	15.71	0.06	0.88

^a^Assessed with 11 replicates tested on the same day.

^b^Assessed with 2 replicates tested on 32 different days (n = 64).

^c^Cycle Threshold.

**Fig 2:**
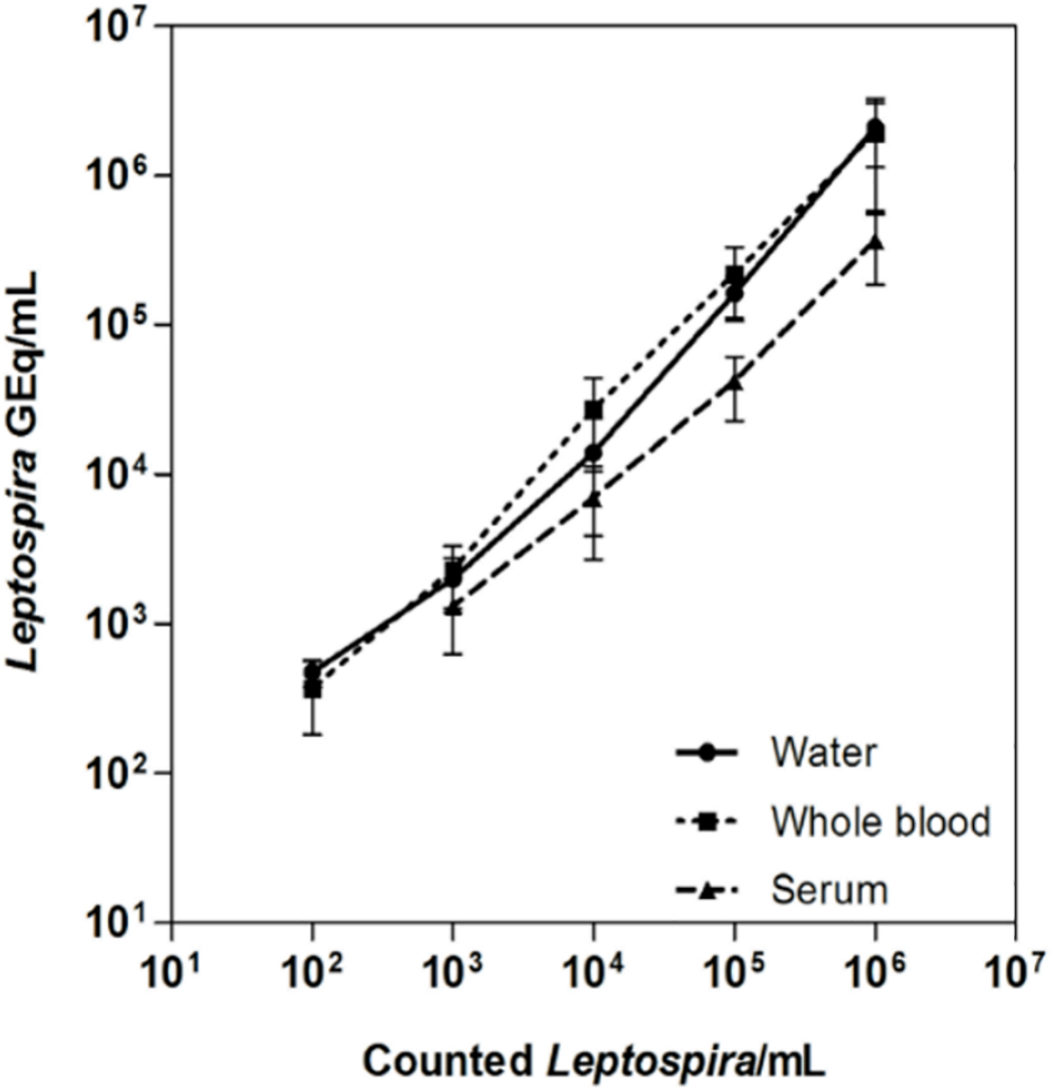
Effect of different matrices on the accuracy of the *lipL32* real-time PCR test. Microscopically counted leptospires were used to spike EWB, serum and ultrapure water to 10^6^ leptospires/mL. Samples were diluted ten-fold serially and the respective DNA extracts were quantified by real-time PCR. Each point represents the mean result of four spiking experiments performed on water, whole blood and serum. Error bars show the mean ± SD.

Linear regression showed that the assay was highly accurate (slope = 0.9396; y-intercept = 0.5745) (Fig 2). Comparison of slopes and y-intercepts of the best-fit lines calculated from the spiking experiments showed that when compared to spiked water the effect of sample matrix on PCR efficiency was significant for serum (*p* <0.0001) but not for whole blood (*p* = 0.2603). The present real-time PCR assay had a limit of detection of 1 × 10^3^ GEq/mL in spiked serum samples (Fig 2).

### Diagnostic accuracy

**Fig 3:**
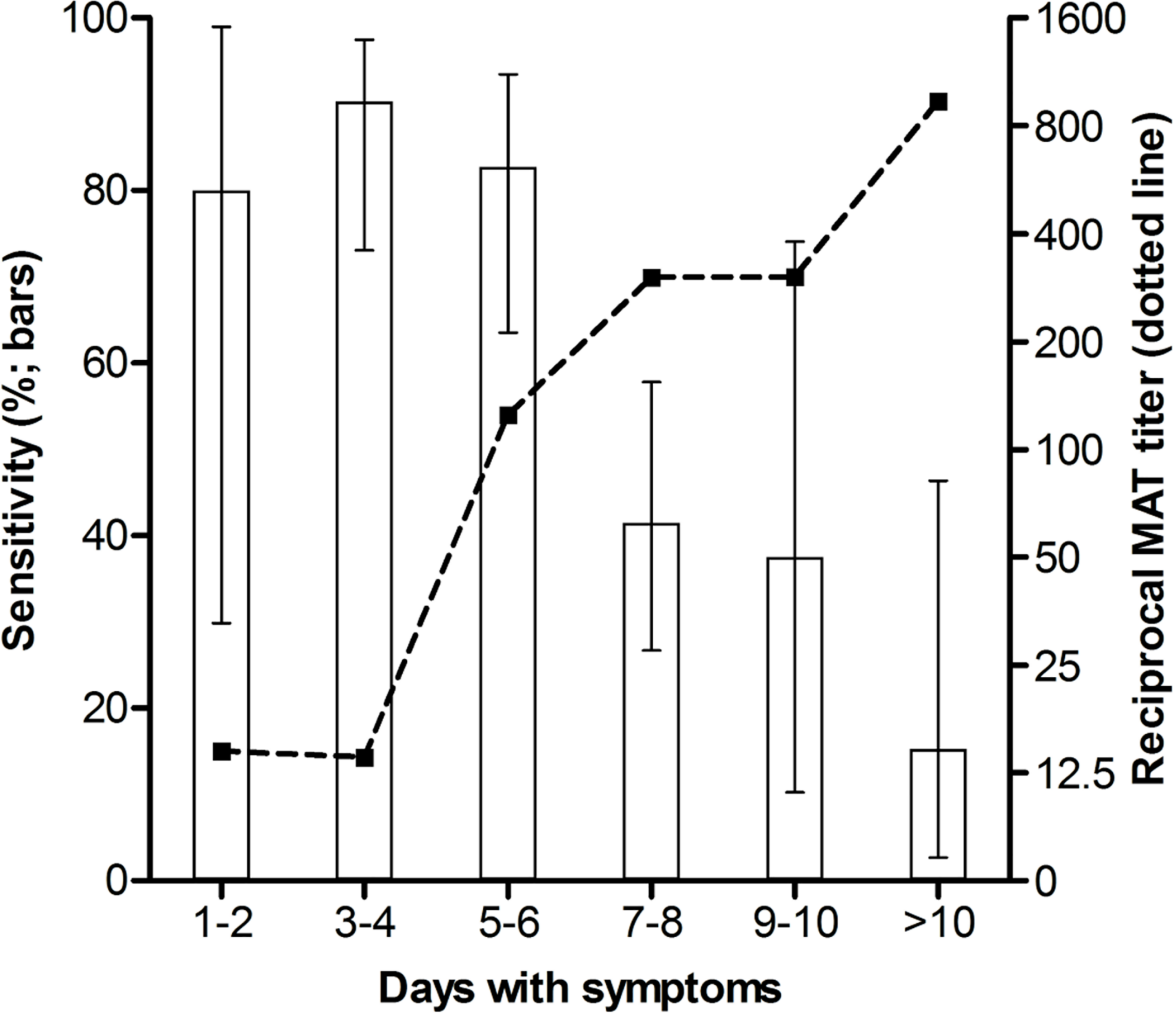
Positivity of the *lipL32* real-time PCR assay according to days after onset of symptoms in suspected cases of leptospirosis. The bars represent the percentage of positive whole blood samples stratified according to days with symptoms (error bars show the 95% CI). The dashed line shows the geometric mean of the reciprocal MAT titer, in accordance with days with symptoms.

Demographic and clinical characteristics of the suspected cases are detailed in Table 2. Since the difference between the real-time PCR results obtained for samples from Salvador and Curitiba was not statistically significant, data were grouped for analysis and are summarized in Table 3. Among the 127 confirmed cases, the sensitivity of the real-time PCR assay was 60.6% (95% CI 51.5–69.1) and 29.1% (95% CI 21.6–38.0%) in acute EWB and serum samples, respectively. Clinical sensitivity was higher in EWB samples (86.1%; 95% CI 74.8–93.1%) than in serum samples (43.1%; 95% CI 31.1–55.9%), within the first six days of illness. Results showed that real-time PCR positivity peaked when patients had symptoms for 3–4 days, then progressively decreased along time and was inversely correlated to the increase in reciprocal MAT titer (Fig 3). As expected, patients who were positive by real-time PCR (n = 87) reported fewer symptomatic days at admission than those who had a negative PCR result (5.2 ± 0.3 vs. 8.7 ± 0.7; *p* <0.0001).

**Table 2:** Characteristics of confirmed cases of leptospirosis. Patients were identified during active surveillance in two Brazilian cities (Salvador and Curitiba), obtained on hospital admission.

	Salvador (n = 85)		Curitiba (n = 42)		Total (n = 127) ^a^	
	n^b^	Mean (SD) or %	n^b^	Mean (SD) or %	n^b^	Mean (SD) or %
Age (years)	85	34.2 (14.5)	42	34.9 (16.1)	127	34.5 (15.0)
Male	85	89.4	42	85.7	127	88.2
Days of illness	84	6.3 (5.0)	42	7.7 (4.4)	126	6.8 (4.8)
Fever	85	95.3	42	90.5	127	93.7
Conjuntival suffusion	85	14.1	42	16.7	127	15.0
Leukocyte count (×10^9^/L)	82	14.5 (7.4)	42	12.0 (5.9)	124	13.7 (7.0)
Jaundice	85	78.8	42	73.8	127	78.0
Total serum bilirrubin (mg/dL)	47	11.4 (9.5)	38	11.5 (11.7)	85	11.5 (10.5)
Oliguria	85	25.9	42	26.2	127	26.0
Serum creatinine (mg/dL)	84	5.6 (12.1)	40	5.3 (7.8)	124	5.5 (10.9)
Blood urea nitrogen (mg/dL)	85	43.8 (41.9)	40	43.9 (35.2)	125	43.8 (39.8)

^a^The mean difference of characteristics of patients from Salvador and Curitiba was assessed by either the Student’s unpaired *t* test or chi-square test and found not to be statistically significant.

^b^Number of responses.

**Table 3:** Diagnostic performance of the real-time PCR detection assay for confirmed and unconfirmed cases of leptospirosis and control subjects.

Subjects	No.	Whole Blood^a^	Serum^a^	Culture^a^	MAT^a^
*Confirmed cases*^b^	127	60.6 (51.5–69.1)	29.1 (21.6–38.0)	11.8 (7.0–19.0)	89.8 (82.8–94.2)
1–2 days of illness	5	80.0 (29.9–98.9)	40.0 (7.2–82.9)	60.0 (17.0–92.7)	20.0 (1.0–70.1)
3–4 days of illness	31	90.3 (73.1–97.5)	48.4 (30.5–66.6)	19.3 (8.1–38.0)	90.3 (73.1–97.5)
5–6 days of illness	29	82.7 (63.5–93.5)	37.9 (21.3–57.6)	17.2 (6.5–36.5)	87.1 (69.2–95.8)
7–8 days of illness	41	41.5 (26.7–57.8)	19.5 (9.3–35.4)	2.4 (0–14.4)	92.7 (80.0–98.1)
9–10 days of illness	8	37.5 (10.2–74.1)	12.5 (0.6–53.3)	0 (0–0.4)	100.0 (59.8–100.0)
>10 days of illness	13	15.3 (2.7–46.3)	0 (0–0.3)	0 (0–0.3)	91.7 (59.7–99.6)
*Unconfirmed cases*^c^	23	43.5 (23.9–65.1)	13.0 (3.4–34.7)	0 (0–0.2)	0 (0–0.2)
Single sample	18	55.6 (31.3–77.6)	22.2 (7.4–48.1)	0 (0–0.2)	0 (0–0.2)
Paired samples	5	0 (0–0.4)	0 (0–0.4)	0 (0–0.4)	0 (0–0.4)
*Control Subjects*	182	1.1 (0.2–4.3)	0 (0–2.6)	0 (0–2.6)	0 (0–2.6)
Other febrile diseases	122	1.6 (0.3–6.4)	0 (0–3.8)	0 (0–3.8)	0 (0–3.8)
Blood bank donors	60	0 (0–7.5)	0 (0–7.5)	0 (0–7.5)	0 (0–7.5)

^a^ Results are presented as % positive (95% CI).

^b^Laboratory confirmation was based on the results of either MAT or culture.

^c^Unconfirmed cases were stratified according to the availability of either acute samples or paired acute and convalescent samples for serological laboratory confirmation. Probable cases were included in this group.

All 15 confirmed cases who were culture-positive also had a positive real-time PCR result when EWB samples were analyzed. However, 8/15 respective serum samples (53.3%, 95% CI 24.7–77.7%) had a positive real-time PCR result. Samples from culture-positive patients yielded significantly lower Ct values than culture-negative cases (33.2 ± 0.6 vs 36.7 ± 0.3; *p* <0.0001), when EWB samples were analyzed.

A total of 12/150 (8.0%) clinically suspected cases died due to complications of leptospirosis, all of whom were real-time PCR positive when EWB samples were analyzed. In contrast, only 8/12 (66.6%) serum samples led to the detection of *Leptospira* DNA. Ct values observed for EWB samples from fatality cases were significantly lower than those observed for survivors (33.6 ± 0.8 vs. 36.5 ± 0.3; *p* <0.0001). Twenty-two of the 150 (14.6%) suspected cases (17 from Salvador and 5 from Curitiba) had clinical evidence of Leptospirosis-associated Pulmonary Hemorrhagic Syndrome (LPHS), as detailed in Table 4. Among those, 20 (91.0%) were confirmed leptospirosis cases, of whom 6 (30.0%) died and 17 (85.0%) had a positive EWB real-time PCR result. Real-time PCR also detected *Leptospira* DNA in EWB in one of the two (50.0%) LPHS non-confirmed cases. This positive patient did not have a convalescent sample analyzed and died due to complications of the disease. The four patients with negative real-time PCR results (3 confirmed, 1 non-confirmed) had symptoms for eight or more days, a milder form of LPHS and survived the disease.

**Table 4:** Real-time PCR assay results according to leptospirosis clinical outcome and sample type.

Clinical Outcome	Days with symptoms Mean (SD)	No. patients (Fatalities)	Real-time PCR result			
			EWB^a^		Serum	
			No. positives	Mean Ct^b^ (SD)	No. positives	Mean Ct^b^ (SD)
*LPHS*^c^	5.4 (2.9)	22 (7)	18	34.9 (2.3)	11	36.8 (1.8)
Confirmed cases	4.8 (2.0)	20 (6)	17	34.9 (2.3)	11	36.8 (1.8)
Non-confirmed cases	11.0 (5.7)	2 (1)	1	36.9 (0)	0	0
*Non-LPHS*	6.7 (4.8)	128 (5)	69	36.4 (2.7)	29	37.0 (2.4)
Confirmed cases	6.5 (2.7)	107 (4)	60	36.3 (2.8)	26	36.9 (2.4)
Probable cases	7.0 (1.4)	2 (1)	2	33.1 (0)	1	37.8 (0)
Non-confirmed cases^d^	4.8 (3.3)	19 (0)	7	37.8 (1.4)	2	39.8 (0.1)
Single sample	4.1 (2.8)	15 (0)	7	37.8 (1.4)	2	39.8 (0.1)
Paired sample	7.7 (3.9)	4 (0)	0	0	0	0

^a^EDTA whole blood.

^b^Cycle threshold.

^c^Leptospirosis-associated Pulmonary Hemorrhagic Syndrome.

^d^Non-confirmed cases were stratified according to the availability of either acute samples or paired acute and convalescent samples for serological laboratory confirmation.

For 18 of the 23 (78.3%) non-confirmed or probable cases, laboratory diagnosis was based solely on the analysis of an acute sample (average days with symptoms, 6.4; Table 3). EWB samples from 10/18 cases (56.0%) were real-time PCR positive, including the two (11.0%) that were probable cases. In contrast, none of the five cases for which leptospirosis diagnosis was excluded after analysis of a convalescent paired sample were positive by real-time PCR. Among the 122 controls diagnosed with an AFI other than leptospirosis, two (1.6%) had a positive real-time PCR result on EWB samples while none of the 60 healthy controls were positive by real-time PCR, resulting in an overall diagnostic specificity of 98.0%.

## Discussion

To date, numerous molecular tests have been developed aiming at the early diagnosis of infectious diseases, including those caused by fastidious organisms such as *Leptospira*. Probe-based real-time PCR provides advantages over the conventional reference methods used to diagnose leptospirosis such as reduced turnaround and hands-on time, low carryover contamination risk and higher sensitivity and specificity. In this study, we report the diagnostic accuracy evaluation of a real-time PCR method for the detection of pathogenic leptospires in human clinical samples.

Herein, a previously described real-time PCR method [16] was optimized to be performed in a different instrument, ensuring the proper diagnostic performance. The LLOD calculated by probit analysis was 2.8 GEq/reaction, which is highly similar to the 3.0 GEq/reaction lowest theoretically possible LLOD for real-time PCR quantitative methods [29] and is equivalent to prior reports [10–13, 15, 19, 23, 31, 32]. When the DNA extraction and PCR amplification protocols used in this study are taken into account, detection of 2.8 GEq/reaction equals a concentration of 280 leptospires per mL of sample, assuming the DNA extraction is 100% efficient. However, the LLOD determined by spiking of healthy blood donor samples may not be extrapolated to diagnostic specimens due to PCR inhibitors potentially present in clinical samples [31]. Since analytical specificity has been previously determined for the oligonucleotides used in this study [16, 23], re-evaluation was not necessary. It is important to note that the oligonucleotides evaluated herein specifically target pathogenic *Leptospira* species. Thus, it is possible that infections caused by intermediate species could be potentially missed. In addition, the assessed analytical parameters (linear dynamic range, analytical sensitivity and specificity, precision and trueness) met the general requirements established for diagnostic in-house real-time PCR assays [33].

In the present study, DNA extracted from EWB yielded a higher sensitivity for the detection of *Leptospira* than DNA extracted from serum samples. This finding was observed for spiked and clinical samples and is corroborated by previous studies [16, 31, 34–36]. Spiked serum samples consistently yielded lower leptospiral DNA concentrations than the nominal input, whereas no matrix effect was observed upon comparison of the results obtained from spiked water and EWB samples. Results from the diagnostic accuracy evaluation support this observation since 60.6% of the confirmed cases rendered positive real-time PCR results upon analysis of EWB samples, when compared to 29.1% positivity for the equivalent serum samples. Taken together, those results indicate that EWB is a better sample for real-time PCR studies. A reason for the higher leptospiral DNA positivity in whole blood may be the internalization of leptospires by circulating leukocytes [16, 36]. Moreover, leptospires have been described as cell-adherent bacteria and could be retained in the clot [32], impairing the detection of low leptospiral DNA concentrations in serum samples. Two of the 122 controls diagnosed with an AFI other than leptospirosis had a positive qPCR result. These two patients were recruited in Salvador and diagnosed with dengue. Leptospirosis was excluded based on culture and MAT results. However, since both leptospirosis and dengue are endemic in Salvador and both patients had less than 7 days with symptoms, it is possible that they were infected with both pathogens as has been previously demonstrated in other locations [37, 38].

According to the findings of the present study, the likelihood of a real-time PCR false-negative result increases if leptospirosis patients took more than six days to seek medical attention and have a blood sample drawn. It is known that leptospiral load reaches the maximum level during the first days of illness and then progressively decreases until becoming negative as antibody titers increase due to functional organization of the host immune response [1, 12]. Consequently, optimal diagnostic windows differ for molecular and serological methods through the course of the disease [6]. Confirmed patients enrolled in this study had on average 6.8 days with symptoms, but those who were detected by real-time PCR had fewer days with symptoms on admission than those who were negative. *Leptospira* DNA was detected in 10 (55.5%) of the 18 non-confirmed cases whose diagnosis was based on the analysis of a single acute sample. However, real-time PCR was negative for the five non-confirmed cases that had both acute and convalescent samples analyzed. Based on these observations, it would be expected that if a convalescent serum sample was available for the 10 non-confirmed cases that had a real-time PCR positive result in an acute sample, seroconversion would potentially be detected by other testing methods. This hypothesis is supported by previous findings [22, 39] and, while both culture and MAT reference tests have limitations [40], might thus introduce a bias in the evaluation of the clinical performance of molecular methods. Nevertheless, the overall diagnostic accuracy was comparable to previous reports of molecular detection of leptospires in EWB samples [12, 15, 19, 23].

All the patients from whose samples leptospires were isolated were also positive by real-time PCR. Additionally, 72 patients were shown to be leptospiremic, but with a negative culture result. Such results confirmed the low diagnostic sensitivity of culture and demonstrated the real-time PCR suitability for early diagnosis. These data also suggest that real-time PCR could be used to drive culture inoculation, if heparin-anticoagulated whole blood samples are available [9]. In this situation, culture media would only be inoculated with samples that were previously positive when tested by real-time PCR. This approach would significantly reduce labor and costs associated with the isolation of leptospires.

In summary, this study describes the performance testing and diagnostic accuracy assessment of a previously described real-time PCR assay aiming at its clinical validation for early diagnosis of human leptospirosis. The assay is technically reliable for the detection of pathogenic leptospiral DNA in EWB samples, with a better performance when compared to serum samples. In addition, we demonstrated the early detection of leptospires in EWB samples from all patient deaths and severe cases who presented within the first six days with symptoms. An external clinical validation involving repeated studies in similar populations presenting variable endemicity levels would allow the assessment of the test actionability in different settings.

## Supporting information

S1 Table *Leptospira* reference strain panel used for the micro agglutination test (MAT). (DOCX)

S2 Table Standards for Reporting of Diagnostic Accuracy (STARD) adherence checklist. (DOCX)

S1 Fig Linearity of the *lipL32* real-time PCR assay. The linear dynamic range was determined by regression analysis using a calibration curve with concentrations ranging from 1 × 10^7^ to 1 × 10^0^ GEq/reaction. Each point represents the mean of 2 replicates tested on 32 different days (n = 64). Error bars represent the geometric mean ± SD.
(TIF)
