# Supplementary material for: Rapid, actionable diagnosis of urban epidemic leptospirosis using a pathogenic Leptospira lipL32-based real-time PCR assay
Source: PLoS Negl Trop Dis. 2017 Sep 15;11(9):e0005940. doi: 10.1371/journal.pntd.0005940 (PMC5617227; doi:10.1371/journal.pntd.0005940)
Supplement: S1 Table — (DOCX) [file pntd.0005940.s001.docx]

**S1 Table. *Leptospira* reference strain panel used for the Microagglutination Test (MAT).**

| Genomospecies | Serovar | Strain |
| --- | --- | --- |
| *Leptospira interrogans* |  |  |
|  | Australis | Ballico |
|  | Autumnalis | Akiyami A^a^ |
|  | Bataviae | Van Tienem |
|  | Bratislava | Jez-Bratislava |
|  | Canicola | Hond Utrecht IV^a^ |
|  | Copenhageni | M20^a^ |
|  | Copenhageni | L1-130^b^ |
|  | Djasiman | Djasiman |
|  | Hardjo | Hardjioprajitno |
|  | Hebdomadis | Hebdomadis |
|  | Icterohaemorrhagiae | RGA |
|  | Pomona | Pomona |
|  | Pyrogenes | Sallinem |
|  | Saxkoebing | Mus 24 |
|  | Wolffi | 3705 |
| *Leptospira biflexa* |  |  |
|  | Patoc | Patoc 1 ^b^ |
| *Leptospira borgpetersenii* |  |  |
|  | Ballum | Mus 127 ^b^ |
|  | Javanica | Veldrat Batavia 46 |
|  | Sejroe | M84 |
|  | Tarassovi | Perepelitsin |
| *Leptospira kirschneri* |  |  |
|  | Cynopteri | 3522 C |
|  | Grippotyphosa | Moskva V^a^ |
| *Leptospira noguchii* |  |  |
|  | Panama | CZ 214 K |
| *Leptospira santarosai* |  |  |
|  | Shermani | LT 821 |
| *Leptospira weilii* |  |  |
|  | Celledoni | Celledoni |

^a^Strains used in Curitiba and Salvador.

^b^ Strains used only in Salvador.
