# Supplementary material for: Rapid, actionable diagnosis of urban epidemic leptospirosis using a pathogenic Leptospira lipL32-based real-time PCR assay
Source: PLoS Negl Trop Dis. 2017 Sep 15;11(9):e0005940. doi: 10.1371/journal.pntd.0005940 (PMC5617227; doi:10.1371/journal.pntd.0005940)
Supplement: S2 Table — (DOCX) [file pntd.0005940.s002.docx]

S2 Table. **Standards for Reporting of Diagnostic Accuracy (STARD) adherence checklist.**

| **Section and Topic** | **No** | **Item** | **Reported on page #** |
| --- | --- | --- | --- |
| **TITLE OR ABSTRACT** | | | |
|  | **1** | Identification as a study of diagnostic accuracy using at least one measure of accuracy (such as sensitivity, specificity, predictive values, or AUC) | Page #2 |
| **ABSTRACT** | | | |
|  | **2** | Structured summary of study design, methods, results, and conclusions  (for specific guidance, see STARD for Abstracts) | Page #2 |
| **INTRODUCTION** | | | |
|  | **3** | Scientific and clinical background, including the intended use and clinical role of the index test | Pages #4-5 |
|  | **4** | Study objectives and hypotheses | Page #5 |
| **METHODS** | | | |
| *Study design* | **5** | Whether data collection was planned before the index test and reference standard were performed (prospective study) or after (retrospective study) | Page #5 |
| *Participants* | **6** | Eligibility criteria | Pages #5-6 |
|  | **7** | On what basis potentially eligible participants were identified  (such as symptoms, results from previous tests, inclusion in registry) | Page #6 |
|  | **8** | Where and when potentially eligible participants were identified (setting, location and dates) | Pages #5-6 |
|  | **9** | Whether participants formed a consecutive, random or convenience series | Page #5 |
| *Test methods* | **10a** | Index test, in sufficient detail to allow replication | Pages #8-9 |
|  |  |  |  |
|  | **10b** | Reference standard, in sufficient detail to allow replication | Page #6 |
|  | **11** | Rationale for choosing the reference standard (if alternatives exist) | Page #6 |
|  | **12a** | Definition of and rationale for test positivity cut-offs or result categories  of the index test, distinguishing pre-specified from exploratory | Page #9 |
| **Section and Topic** | **No** | **Item** | **Reported on page #** |
|  | **12b** | Definition of and rationale for test positivity cut-offs or result categories  of the reference standard, distinguishing pre-specified from exploratory | Page #6 |
|  | **13a** | Whether clinical information and reference standard results were available  to the performers/readers of the index test | Page #8 |
|  | **13b** | Whether clinical information and index test results were available  to the assessors of the reference standard | Page #6 |
| *Analysis* | **14** | Methods for estimating or comparing measures of diagnostic accuracy | Pages #9-10 |
|  | **15** | How indeterminate index test or reference standard results were handled | Page #20 |
|  | **16** | How missing data on the index test and reference standard were handled | Not Applicable |
|  | **17** | Any analyses of variability in diagnostic accuracy, distinguishing pre-specified from exploratory | Pages #10-13 |
|  | **18** | Intended sample size and how it was determined | Not Applicable |
| **RESULTS** | | | |
| *Participants* | **19** | Flow of participants, using a diagram | Figure 1 |
|  | **20** | Baseline demographic and clinical characteristics of participants | Table 2 |
|  | **21a** | Distribution of severity of disease in those with the target condition | Pages #18-20; Table 4 |
|  | **21b** | Distribution of alternative diagnoses in those without the target condition | Pages #18, 20 |
|  | **22** | Time interval and any clinical interventions between index test and reference standard | Page #6 |
| *Test results* | **23** | Cross tabulation of the index test results (or their distribution)  by the results of the reference standard | Table 3 |
|  | **24** | Estimates of diagnostic accuracy and their precision (such as 95% confidence intervals) | Pages #10-13; Table 1 |
| **Section and Topic** | **No** | **Item** | **Reported on page #** |
|  | **25** | Any adverse events from performing the index test or the reference standard | Not Applicable |
| **DISCUSSION** | | | |
|  | **26** | Study limitations, including sources of potential bias, statistical uncertainty, and generalizability | Pages #21-22 |
|  | **27** | Implications for practice, including the intended use and clinical role of the index test | Page #23 |
| **OTHER INFORMATION** | | | |
|  | **28** | Registration number and name of registry | Supplementary Information |
|  | **29** | Where the full study protocol can be accessed | Supplementary Information |
