# Supplementary figures and images for: Rapid, actionable diagnosis of urban epidemic leptospirosis using a pathogenic Leptospira lipL32-based real-time PCR assay
Source: PLoS Negl Trop Dis. 2017 Sep 15;11(9):e0005940. doi: 10.1371/journal.pntd.0005940 (PMC5617227; doi:10.1371/journal.pntd.0005940)

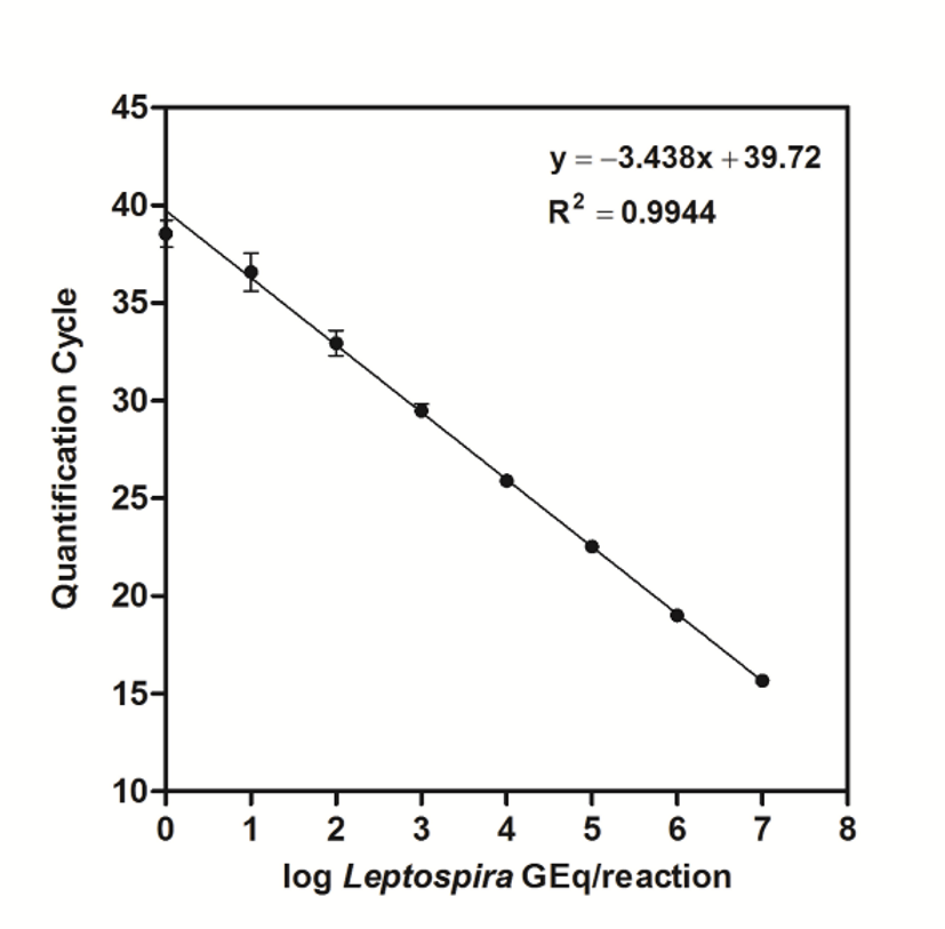

Supplement: S1 Fig — The linear dynamic range was determined by regression analysis using a calibration curve with concentrations ranging from 1 × 10^7^ to 1 × 100 GEq/reaction. Each point represents the mean of 2 replicates tested on 32 different days (n = 64). Error bars represent the geometric mean ± SD. (TIF) [file pntd.0005940.s003.tif]
